# Behavioral and neurophysiological effects of buspirone in healthy and depression-like state juvenile salmon

**DOI:** 10.3389/fnbeh.2024.1285413

**Published:** 2024-02-12

**Authors:** Sheyda Shapouri, Aziz Sharifi, Ole Folkedal, Thomas W. K. Fraser, Marco A. Vindas

**Affiliations:** ^1^Biochemistry and Physiology Unit, Faculty of Veterinary Medicine, Norwegian University of Life Sciences, As, Norway; ^2^Department of Biosciences, Faculty of Mathematics and Natural Sciences, University of Oslo, Oslo, Norway; ^3^Animal Welfare, Matre Research Station, Institute of Marine Research, Bergen, Norway; ^4^Reproduction and Developmental Biology, Matre Research Station, Institute of Marine Research, Bergen, Norway

**Keywords:** depression models, behavioral inhibition, serotonergic activity, chronic stress, cortisol

## Abstract

A proportion of farmed salmon in seawater show a behaviorally inhibited, growth stunted profile known as a depression-like state (DLS). These DLS fish are characterized by chronically elevated serotonergic signaling and blood plasma cortisol levels and the inability to react further to acute stress, which is suggestive of chronic stress. In this study, we characterize the neuroendocrine profile of growth stunted freshwater parr and confirm that they show a DLS-like neuroendocrine profile with a blunted cortisol response and no serotonergic increase in response to acute stress. Furthermore, we attempted to reverse this DLS-like profile through pharmacological manipulation of the serotonin (5-HT) system with buspirone, an anxiolytic medication that acts as a serotonin receptor agonist (i.e., decreases serotonergic signaling). We found that while buspirone decreases anxiolytic-type behavior in healthy fish, no quantifiable behavioral change was found in DLS-like fish. However, there was a physiological effect of diminished basal serotonergic signaling. This suggests that at the physiological level, buspirone appears to reverse the neuroendocrine DLS profile. With a deeper understanding of what causes DLS profiles and growth stunting in juvenile fish, steps can be taken in terms of husbandry to prevent repeated stressors and the formation of the DLS profile, potentially reducing losses in aquaculture due to chronic stress.

## Introduction

Atlantic salmon (*Salmo salar*) have been steadily increasing as a research model in recent years. This is partly due to it being a culturally and economically important species, as well as being of great interest in behavioral and evolutionary ecology due to their complex life cycle (Hoar and Randall, 1988; Liu et al., 2011; Finn, 2020). Farmed Atlantic salmon are undergoing a rapid domestication process and are faced with extraordinarily intensive production environments, which can be highly stressful for some individuals. We have previously reported that a proportion of farmed salmon in the seawater stage are characterized by a depression-like state (DLS): small, thin, and easily catchable at the surface; exhibiting anorexia and a behaviorally inhibited profile. Furthermore, DLS fish show both chronically elevated levels of the stress hormone cortisol and signaling of the neurotransmitter serotonin (hydroxytryptamine; 5-HT) in the brain stem at basal levels, and the inability to respond to stress with increased 5-HT and cortisol levels as occurs in healthy salmon (Vindas et al., 2016). Neural signaling systems activated under stress are highly conserved (Winberg and Nilsson, 1993; Lillesaar, 2011; Herculano and Maximino, 2014), suggesting adaptive effects of their function. However, pathologies may evolve from a mismatch between the historic and current environment (Nettle, 2004). In this context, Vindas et al. (2016) suggested that DLS salmon are experiencing a mismatch to their current aquaculture environment from their natural one and are thus unable to cope. Notably, this DLS profile has only been characterized in seawater and it is unknown if it is already present during the freshwater stages in juvenile parr. Furthermore, it is yet unknown if these DLS profile is reversible.

The purpose of this study is to test the hypothesis that growth stunted parr show a DLS-like neuroendocrine profile. Furthermore, we further hypothesized that it is possible to reverse this DLS-like profile by decreasing 5-HT activity with buspirone, a drug with anxiolytic effects, which acts via 5-HT_1A_ receptors decreasing the amount of 5-HT in the synaptic cleft, which reduces serotonergic signaling (Gebauer et al., 2011). We first conducted a dose response experiment to determine the buspirone concentration, dosage, and method of administration on Atlantic salmon. Following this, DLS-like fish were repeatedly treated with buspirone over 4 weeks. We quantified behavior, monoamine neurochemistry and gene expression to understand where and how 5-HT manipulation affects the DLS-like profile. With this, we aimed at elucidating the mechanism behind the regulation of the DLS profile. This knowledge will give us a broader understanding of DLS in vertebrates as well as help us determine practical solutions (such as diminishing stress challenges) which may help reduce the chronic elevation of 5-HT in aquaculture environments.

## Materials and methods

### Ethics statement

The experiments were performed in accordance with current Norwegian law for experimentation and procedures on live animals, and was approved by the Norwegian Food Safety Authority (Mattilsynet) through FOTS application ID 18619.

### Dose response experiment

#### Experimental facilities and fish

The dose response experiment was performed at the Norwegian University of Life Sciences (NMBU) veterinary facilities in Oslo, Norway, in June of 2019 over a period of 10 days. All fish were obtained from the salmon fish facilities at the Norwegian University of Life Sciences, in Ås, Norway. The fish were reared at this fish facility in indoor experimental tanks (Ø = 3 m, volume = 7 m^3^) at densities between 3 and 10 kg/m^3^ (from start feeding to parr stage) on a 24-h light regime, with water temperatures ranging from 13.4 to 15.1°C and *ad libitum* food, following established routines by the university. The water quality parameters were maintained within husbandry standards for Atlantic salmon, with an alkalinity between 1.2 and 1.5, pH between 7.3 and 7.8, NO_3_ between 20.1 and 30.2 mg/L, and a flow of approximately 15 L/min.

#### Experimental design

The dose response experiment was performed to adequately judge dosing of buspirone, as well as the method for administering it to groups of juvenile Atlantic salmon kept in freshwater, and it was divided into two parts. A total of 12 juvenile salmon with an approximate weight of 130 g were brought to the laboratory facilities at NMBU. The stress from transport by car and into glass aquaria, a novel environment, contributed to these fish experiencing fearful and behaviorally inhibited states. The 12 fish were separated into groups of four in three aquaria (100 × 50 × 50 cm) set up in a row with wireless CCTV cameras (Foscam FI9851P, Egnir Invest, Son, Norway) directed horizontally at the aquaria, with the video feed controlled remotely by a computer in a different room to minimize disruption.

The fish were kept on 24 h light, and air stones provided aeration and ensured 85–95% total oxygen saturation throughout the experiment. The water temperature was between 13 and 15°C. All tanks were filled with 250 L dechlorinated Oslo tap water with a pH of 7 and NO_3_ levels 10 mg/L. In addition, 50% water changes were performed approximately every 2 days in order to maintain high water quality standards.

##### Dose response experiment part 1

The fish were assigned to three different treatments: control (aquarium 1), a 3 mg/L buspirone concentration (aquarium 2), and a 5 mg/L buspirone concentration (aquarium 3). These buspirone concentrations were initially diluted in 5 mL Oslo tap water and the concentrations were determined by previously reported effects of buspirone on fish (Bencan et al., 2009). The concentrations were administered by lowering diluted buspirone into the aquaria in small 5 L glass vials. Note that the control aquarium was similarly disturbed by lowering a vial containing only water. All aquaria had static water conditions with air stones providing oxygenation. Video recordings started 10 min prior to the buspirone treatment and continued for 1 h after all aquaria had been treated. After recording had concluded, the fish were left in the treated water for approximately 24 h. At this point we observed that prolonged exposure to buspirone-treated water was harmful, as evidenced by all treated fish showing signs of distress and aberrant behavior. The decision was therefore made to euthanize all 8 treated fish (2 out of the 3 aquaria) with a lethal dose of buffered MS-222 (Finquel^®^, Argent Chemical Laboratories, Redmond, WA, United States) at a concentration of 2 g/L.

##### Dose response experiment part 2

**Figure 1:**
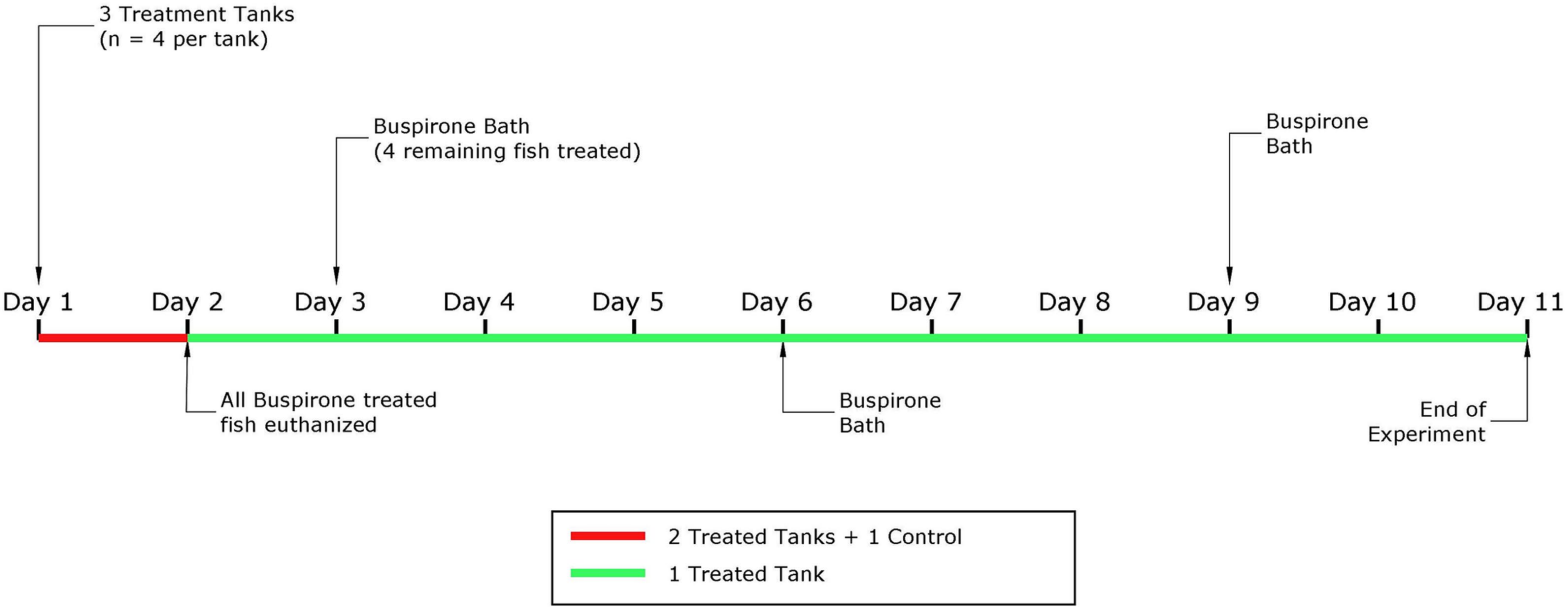
Schematic representation of the experimental timeline for the dose response experiment, with the part 1 highlighted in red and the second part in green.

For this part, we only used one aquarium with 4 fish. Based on the results from part 1, we concluded that the most appropriate buspirone concentration to move forward with for repeated treatment was 3 mg/L. That is, these fish were treated with repeated 3 mg/L buspirone baths at two-day intervals, for 1 h. After treatment, fish were quickly netted and transferred into an adjacent aquarium containing clean water (i.e., no buspirone) to avoid overexposure. In total, this group received three baths over the course of 9 days. Fish were fed 1.5% of their body weight daily in 3 mm dry food pellets (Skretting, Norway) and left undisturbed with the food for 10 min before uneaten food pellets and other debris were siphoned out of the aquarium. Video recording started 10 min prior to buspirone treatment and continued for 1 h after all aquaria had been treated. Figure 1 depicts a timeline explaining both parts of the dose response experiment.

#### Behavioral analysis

Video recordings were analyzed manually by using a stopwatch. The following parameters were quantified: 1. Activity levels: measured as locomotion (i.e., fish moving more than one body length) in 10 min intervals. 2. Cohesion: how close together the fish swam to each other was calculated by measuring the average distance between each fish to every other fish in a picture frame taken every 2 min for a total of 10 min following procedures by Spagnoli et al. (2017). 3. Top and bottom times: the total amount of time fish stayed at the bottom and top half of the aquarium during 10 min intervals. 4. Number of crossings: each time fish crossed between the bottom and top halves of the aquarium during 10 min intervals. The quantification of all behavioral parameters was used as a proxy for anxiety-like behavior (Bencan et al., 2009; Blaser et al., 2010). All behavioral outputs were quantified at three different timepoints: 10 min before (i.e., before), and 10 min after (i.e., after), they were exposed to the buspirone/sham bath, as well as the last 10 min of the 1 h bath (i.e., end). All tests were conducted between 10:00 and 12:00 each day.

### Main buspirone experiment

#### Experimental facilities and fish

The main buspirone experiment was conducted at the Institute of Marine Research (IMR) facility in Matre, Norway. The experiment was performed over the course of 18 days. The fish originated from the domesticated AquaGen SHIELD strain that hatched at the Matre facilities and were kept on 24 h light conditions for approximately 3 months (from start feeding) until the start of the experiment in 3 m Ø 5,500 L circular tanks which were part of a flow through system. They were kept at ambient temperatures (ranging from 14 to 10°C throughout the production period) at a density of 10 kg/L. The water quality parameters were maintained within husbandry standards for Atlantic salmon, with a pH between 6.7 and 7.2 and a flow of approximately 17 L/min.

Farmed salmon are exposed to a series of pathogens throughout their life cycle and are therefore vaccinated before smoltification, when they are around 30–40 g. It is therefore common practice to sort fish before vaccination and discard all fish smaller than 30 g (Koppang et al., 2008). The fish used in this experiment were from the slowest growing fraction (<25 g, ~5% of the fish group) of a group of 40,000 individuals that were sorted before vaccination. These small fish were to be destroyed and therefore, we selected 120 of these small individuals for our experiment. The fish were transferred to opaque tanks in groups of 20. Feeding followed expected growth tables, feed was provided by Skretting AS, and distributed using automatic feeders during the light hours.

In order to characterize the neuroendocrine profile of these small fish in freshwater we conducted an additional sampling on a small group (approximately 3,600 individuals) of fish which were also sorted before vaccination, but during a different production cycle. These fish were produced and kept in the same manner as described above. After sorting the approximately 3,600 fish (<30 g), were put back into their rearing tank (3 m 5,500 L circular tanks). The fish were kept on ambient temperature (14–6°C during the autumn months) and 24 h light for 1 month, before they were changed to a simulated natural photoperiod for 45 days before a subgroup (*n* = 28) were sampled at both basal and post-stress level conditions (*n* = 14 per condition). This group will be further referred to as timepoint 0 (T0), see below for further details.

#### Experimental design

This experiment was conducted on a subset of the sorted parr which were < 30 g. These fish were chosen since they are growth stunted and show low feed intake (i.e., shows symptoms indicative of a DLS profile) during the freshwater stage. The 120 fish (weighing approximately 15 g) were divided into 6 groups of 20 individuals and placed into 6 numbered adjacent opaque tanks. Odd-numbered tanks (1, 3, and 5) were buspirone-treated, and even-numbered tanks (2, 4, and 6) served as control groups. Water flow was standardized across all tanks at 10 L/min, the fish were kept on a photoperiod of 10:14 light/dark at ambient temperatures (60.8760° N, 5.5867° E, 9.4°C on average). Oxygen was maintained at ≥80% saturation and fish were hand fed 1.2 mm pellets twice a day (10:00 and 15:00). All tanks were filmed from above using an automated recording system.

The video feed was displayed on a nearby screen connected to a NoVus multistandard AHD recorder (NHDR-5116AHD, NoVus CCTV, AAT Holding S.A., Warsaw, Poland) that saved all video segments, with all tanks visible simultaneously so the overall behavior could be observed without disturbing the fish. Due to logistical issues with video storage in the AHD recorder, the data for the first 8 days was lost. All video analysis was performed on video selected from the remaining days.

**Figure 2:**
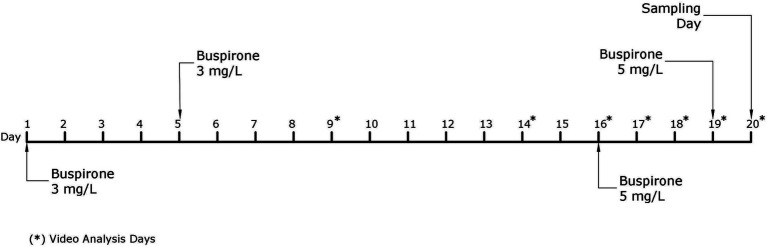
Schematic representation of the experimental timeline for the main buspirone experiment. Days indicated with a star (*) are days in which video analysis of behavioral parameters was performed.

The fish received a total of four baths throughout the course of this experiment, two with a 3 mg/L and two with a 5 mg/L buspirone dose (see discussion for further details regarding the change to a higher buspirone concentration). On bath treatment days, all fish were netted out from their home tanks and placed in 50 L buckets. The buckets were either treated with a vial of dissolved buspirone or a vial of plain water to create an equal disturbance for all groups of fish. After 1 h of treatment fish were quickly netted and returned to their home tanks. Figure 2 depicts a timeline explaining all activities concerning the main buspirone experiment.

#### Sampling

As explained above, 28 fish were sampled directly from their home tank 75 days after sorting (T0). Furthermore, at the end of the experiment, 72 of the 120 experimental fish were also sampled (timepoint 1, T1). All fish were sampled at either basal or post-stress conditions. To test basal conditions, fish (*n_T0_* = 14 and *n_T1_* = 18/treatment) were netted from their home tanks and immediately euthanized. For post-stress conditions, fish (*n_T0_* = 14 and *n_T1_* = 18/treatment) were netted from their home tanks and subjected to a 30-min confinement stress test (following methodology by Vindas et al., 2016). The confinement test consisted of placing individual fish in a 10 L bucket filled with 3 L of water from their home tank, with air stones and pumps maintaining oxygen levels throughout the test. All fish (basal and post-stress groups) were euthanized with a lethal dose of buffered MS-222 (Finquel^®^, Argent Chemical Laboratories, Redmond, WA, United States) at a concentration of 2 g/L until completely unresponsive and motionless (within approximately 30 s) before sampling. Fish were rapidly weighed, fork length measured, and a blood sample was taken from the caudal vessels with a 23G, 1 mL syringes containing the anticoagulant ethylene diamine tetra acetic acid (EDTA). Following centrifugation for 10 min at 9.289 rcf and 4°C, plasma samples were frozen and stored at −80°C for later analysis. Fish were then decapitated and sampled in 2 ways. For the untreated fish, the brains were excised, and the brain stem was placed in an Eppendorf and immediately frozen in dry ice. Meanwhile, for the treated groups, the jaw and gills were trimmed away, the remaining head was then sealed in a plastic bag, snap-frozen on dry ice and stored at −80°C for further processing.

#### Behavioral analysis

The following parameters were quantified: 1. Activity levels: measured as locomotion (i.e., fish moving more than one body length) in 10 min intervals. 2. Cohesion: how close together fish swim to each other was calculated by measuring the average distance between each fish to every other fish in a picture frame taken every 2 min for a total of 10 min following procedures by Spagnoli et al. (2017) (see Supplementary Table S1 for details on number of images and selected timepoints). 3. Aggression: aggressive acts (charging, chasing, nipping and fleeing) were quantified based on parameters described by Keenleyside and Yamamoto (1962), for every alternate min during 10 min for a total of 5 min. Instances of aggression were pooled together and the total number of aggressive acts were used for further analysis (following methodology by Vindas et al., 2012). For the main experiment, the aforementioned behavioral outputs were quantified on days 9, 14, 16, 17, 18, 19, and 20 at approximately 09:00 before the fish were disturbed in any way.

#### Cortisol analysis

Cortisol in plasma from EDTA-treated blood was analyzed using a commercially available DetectX^®^ cortisol enzyme immunoassay kit (Arbor Assays, Ann Arbor, MI, United States), following the manufacturers protocol. The absorbance of the prepared ELISA plate was read in a plate reader at 450 nm and the concentrations were calculated using the four-parameter logistics curve.

#### Brain sectioning and microdissections

A total of 14 brain samples per treatment were randomly selected for processing (*n* = 14 per treatment per condition). However, due to logistical reasons 2 samples from each treatment at basal conditions were lost. Frozen whole heads were sliced in 100 μm serial sections using a Leica CM3050 cryostat (Leica, Wetzlar, Germany) at approximately −23°C. The sliced tissue was thaw-mounted on glass slides, before been refrozen at −80°C.

**Figure 3:**
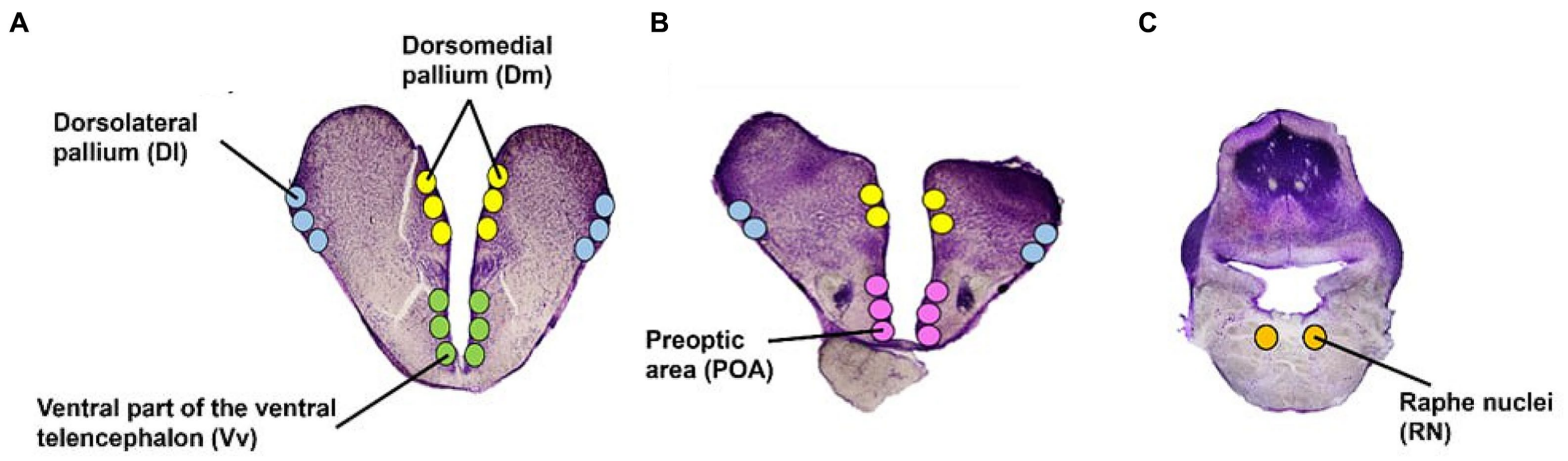
Atlantic salmon brain regions. Transverse view of the five regions of interest in the telencephalon **(A,B)** and brain stem **(C)**. Illustrations have been adapted from several stereotaxic atlases (Carruth et al., 2000; Pérez et al., 2000; Folgueira et al., 2004; Meek and Nieuwenhuys, 2014).

For microdissections, glass slides were kept on an RNase cleaned cold stage set at −14°C. Using a stereo microscope, five areas were microdissected using a 23G needle: the forebrain pallial dorsolateral (Dl) and dorsomedial (Dm) pallium (functionally equivalent to the mammalian hippocampus and amygdala, respectively; Vargas et al., 2009; O’Connell and Hofmann, 2011; Goodson and Kingsbury, 2013), the subpallial ventral part of the ventral telencephalon (Vv); functionally equivalent to the mammalian lateral septum (O’Connell and Hofmann, 2011), the pre-optic area (POA), and the brain stem Raphe Nuclei (RN) (Figure 3). The brain regions were identified using several rainbow trout and salmonid stereotaxic atlases (Carruth et al., 2000; Pérez et al., 2000; Folgueira et al., 2004; Meek and Nieuwenhuys, 2014). The average number of punches (i.e., microdissections from each area) was 24 for the Dl, Dm, and RN; and 12 for the Vv and POA. Microdissected tissue for each area was collected in 100 μL of sodium acetate buffer containing an internal standard (3-4-dihydroxybenzyl amine hydrobromide; DHBA) for monoamine analysis. Subsequently, the samples were placed on dry ice before storage at −80°C.

#### Monoaminergic neurochemistry

The frozen brain stem samples from the T0 groups were first homogenized in 4% (w/v) ice cold perchloric acid (PCA) containing 0.2% EDTA and 3,4-dihydroxybenzyl amine hydrobromide (DHBA, 40 ngmL^−1^) as an internal standard using a Potter–Elvehjem homogenizer. After this, samples were centrifuged for 10 min at 20,000 rcf and 4°C. The supernatant was separated for analyzing monoamine neurochemistry by high-performance liquid chromatography (HPLC).

The frozen samples for the T1 groups were thawed and immediately centrifuged for 10 min at 20,000 rcf and 4°C. The supernatant was separated for analyzing monoamine neurochemistry, while the remaining pellet was dissolved in 350 μL RLT buffer and 3,5 μL 2-beta mercaptoethanol (βME) before it was refrozen at −80°C for further RNA extraction and protein concentration analysis (see next subsections for further details).

The HPLC system consisted of a mobile phase with 86.27 mM sodium dihydrogen phosphate (NaH_2_PO_4_), 3.7 μL ethylenediaminetetraacetic acid (EDTA), and 0.81 mM sodium octyl sulfate (C_8_H_17_NaO_4_S) in deionized water (resistance 18.2 MW) with 7% acetonitrile at pH 3.1. The system was composed of a solvent delivery system (Shimadzu, LC-10 AD), an autoinjector (Famos, Spark), a reverse-phase column (4.6 × 100 mm, Hichrom, C18, 3.5 mm), and an ESA Coulochem II detector (ESA, Bedford, MA, United States) with two electrodes at −40 and + 320 mV. For oxidizing possible contaminants before analysis, a conditioning electrode with a potential of +40 mV was used. Brain 5-HT and its main catabolite 5-hydroxyindoleacetic acid (5-HIAA), were quantified by comparison with standard solutions and corrected for recovery of the internal standard using the Clarity HPLC software (CSW, DataApex Ltd., the Czech Republic).

#### Relative transcript abundance

Prior to RNA extraction, the frozen samples were first thawed and vortexed for 60 s and then homogenized at 5500 RPM for 20 s. From this step and onwards, the total RNA was extracted in accordance with the manufacturer’s instructions for the RNeasy^®^ Plus Mini kit (Qiagen, Crawley, West Sussex, United Kingdom). The flow-through from the RNA extraction was processed for the Bradford protein analysis (see next subsection for further details). RNA concentrations were assessed using Epoch microplate spectrometer (BioTek Instruments, Winooski, VT, United States) and were calculated with the Gen 5 3.00 software (BioTek^®^ Instruments, Inc). RNA quality was inferred from RNA integrity numbers (RINs) calculated using a Bioanalyzer 2100 (Agilent Technologies, Palo Alto, CA, United States). RIN ≥ 5 was considered as acceptable RNA quality (with RIN ≥ 8 indicating excellent quality). Only the Dm and the RN samples had RIN values between 6 and 8, along with the highest average RNA concentrations, and thus were selected for further processing. The cDNA was synthesized from total Dm and RN RNA samples using an iScript cDNA Synthesis Kit (BIO-RAD 1708891) in a total volume of 20 μL. Samples with RNA concentrations less than 1.5 ng/μL were excluded from further analysis.

Gene-specific primers for Atlantic salmon were retrieved from the literature and, are listed in Supplementary Table S2. Calibration curves were run for all primer pairs and qPCR products were sequenced to confirm the specificity of the primers. Quantitative PCR (qPCR) was performed in duplicates using 1 μL of forward and reverse primers in SYBR^®^ GREEN I Master Mix (Roche Diagnostics, Basel, Switzerland) with a 2 μL 1:2 cDNA template. The total reaction volume was 10 μL. The reaction condition for the qPCR was carried out as described by Johansen et al. (2011). Two duplicates with only SYBR^®^ GREEN I Master Mix for each primer pair served as the cDNA negative controls. The quantitation cycle (Cq) values were run on Light Cycler 96 version 1.1.0.1320 (Roche Diagnostics), and the Cq-values and melting curves were analyzed with LightCycler^®^ 96 software (Roche Diagnostics). The reference genes were *s20*, *ef1aα*, *hprt1*, and *ppia*. Since *s20* gave the lowest Cq-values and smallest difference in melting curves both between and within plates, this gene was chosen as the internal control for calculation of relative expression. Samples which had Cq values greater than 35, more than one top in their melting curves and/or technical replicates with an SD difference greater than 0.1 were eliminated following the methodology of qPCR analysis by Bustin et al. (2009). Gene expression levels of the control fish at basal conditions were normalized to 1, and data are presented as normalized values to this treatment control average (fold-change). Notably, since the *5-HT_1Aβ_* gene gave Cq-values >36 and had multiple top melting curves, this gene was excluded from the results.

#### Bradford protein analysis

The flow-through following RNA extraction was treated with 1,400 μL ice-cold acetone and incubated for 30 min at −20°C. After centrifuging for 10 min at 20,000 rcf, the pellets were washed with 100 μL ice-cold ethanol before resuspension in 20 μL 0.4 NaOH buffer. The resuspended pellets were used for Bradford protein assay analysis, as described by Vindas et al. (2014).

### Statistical analysis

RStudio software 4.0.4 (R Development Core Team)^1^ was used for the statistical analysis. The statistical packages “nlme” and “MuMIn” were used for exploratory linear mixed effect (LME) models. The behavioral data for the dose response experiment part 1 was analyzed using an LME, with treatment (control, buspirone 3 and 5 mg/L) and time (before, after and end of the bath) as categorical independent variables and fish as a random effect. Meanwhile, the behavioral data for the dose response experiment part 2 was analyzed using an LME, with time (before, after, end) and day (1, 2, or 3) as categorical independent variables and fish as a random effect. For the T0 group the brain stem monoamine neurochemistry was analyzed using a t test, while the cortisol data was analyzed together with the groups from the main buspirone experiment. For the main buspirone experiment, the behavioral, cortisol, monoaminergic neurochemistry and gene expression data were analyzed using LME models, with treatment (control vs. buspirone) and conditions (basal vs. stress) as categorical independent variables and tank as a random effect. The initial LME models allowed the independent variables to interact. However, the final model was selected based on the lowest Akaike information criterion (AICc) score, i.e., the model with the best data fit when weighted against model complexity, following backwards model building. Visual inspection of the qqnorm and residual plots to check the assumptions of normality and homoscedasticity confirmed that these models conformed to these assumptions (note that *5-HT1Aα* values were square-rooted to achieve homoscedasticity). Interactive effects between treatment and test were assessed using Tukey–Kramer honestly significant difference *post hoc* test. Significance was assessed as *p* ≤ 0.05. Data outliers for both monoamine and gene expression data were determined by the Rosner test (setting the K value at 4) (for more detailed information about the data outliers, see Supplementary Table S3). All data are shown as mean ± SEM.

## Results

### Dose response experiment part 1

#### Locomotion

**Figure 4:**
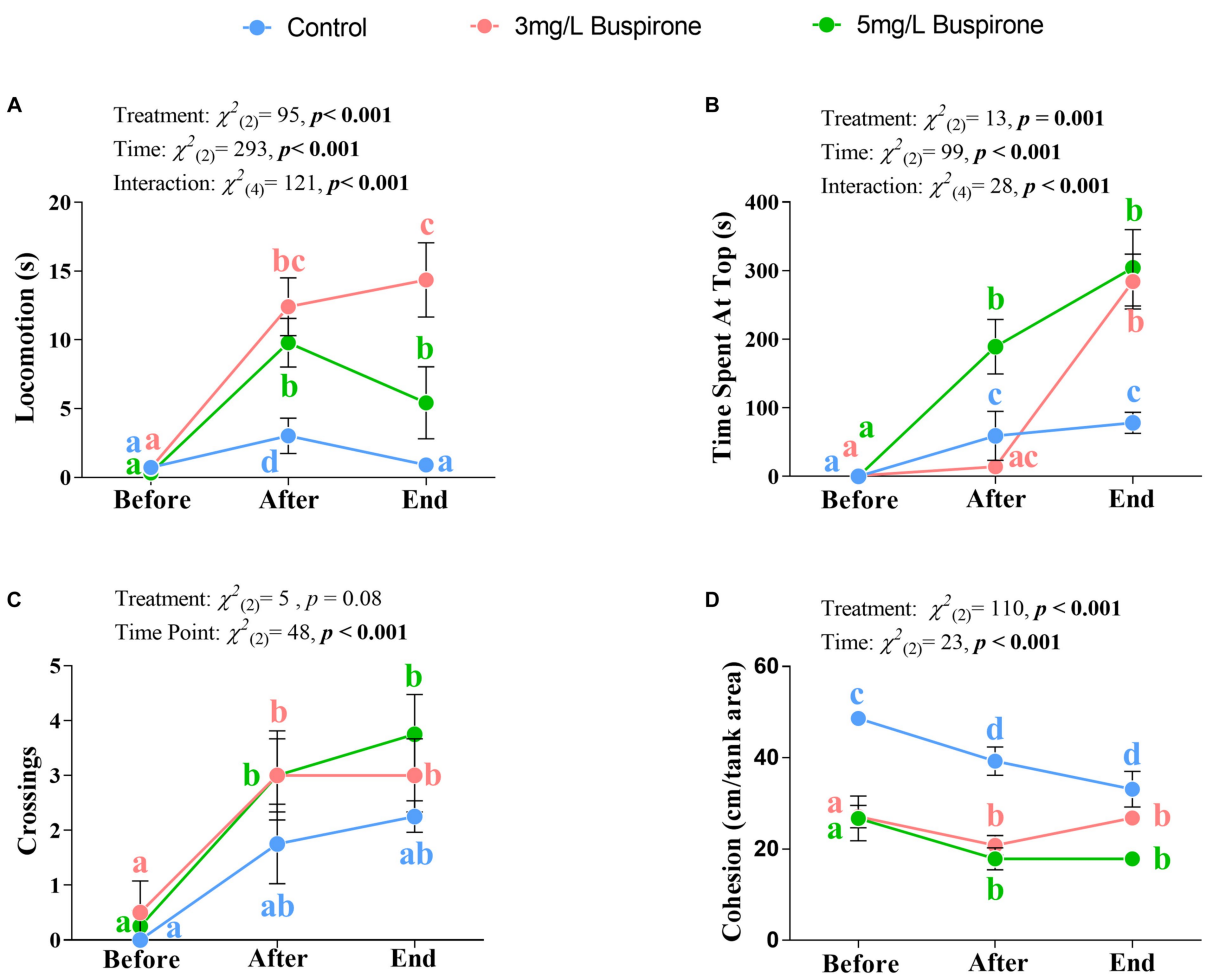
Dose response experiment part 1 (*n* = 4 fish per treatment). Mean ± SEM of locomotion (i.e., time spent moving more than one body length; **(A)**, time spent at the top half of the water column **(B)**, the number of times fish crossed between top and bottom halves **(C)**, and overall group cohesion (measured as average distance between all fish within the group; **(D)** of buspirone-treated (3 and 5 mg/L) and control fish. Measurements were taken 10 min before, 10 min after, and at the last 10 min of the buspirone/sham bath which lasted for 1 h. Linear mixed effect model statistics are given within each panel and small letters symbolize Tukey post-hoc differences.

There were significant effects of treatment [χ^2^_(2)_ = 95, *p* < 0.001], time [χ^2^_(2)_ = 293, *p* < 0.001], and the interaction between treatment and time [χ^2^_(4)_ = 121, *p* < 0.001] on locomotion. The 3 mg/L buspirone lower concentration group showed a significant spike in locomotion between the start and both directly after and at the end of the treatment (*p* < 0.001 for both). Furthermore, the 5 mg/L buspirone concentration group showed a significant increase immediately after treatment (*p* < 0.001) and a significant decline at the end, both compared to before (*p* < 0.001) and after (*p* = 0.005). The control fish had a significant increase in locomotion directly after the sham treatment (*p* = 0.004), which declined at the end of the treatment back to the initial levels. Notably, control groups showed the lowest locomotion levels at both the after and the end timepoints compared to both the 3 mg/L and the 5 mg/L groups (*p* < 0.001 for all). Finally, the 3 mg/L group showed the highest locomotion levels at the end of the treatment compared to both the 5 mg/L and control (*p* < 0.001 for both) groups (Figure 4A).

#### Vertical positioning (time spent at the top)

There were significant effects of treatment [χ^2^_(2)_ = 13, *p* = 0.001], time [χ^2^_(2)_ = 99, *p* < 0.001] and the interaction between treatment and time [χ^2^_(4)_ = 28, *p* < 0.001] on vertical positioning of the fish. Both buspirone treated groups spent significantly more time at the top of the tank at the end, compared to the before time (*p* < 0.001 for both). Furthermore, the 5 mg/L treatment also showed a significant increase in the use of the top between the before and after treatment times (*p* < 0.001). Meanwhile, control groups showed a significant increase in the use of the top of the tank between the before and the after (*p* = 0.01) and the before and end timepoints (*p* < 0.001). Notably, both treatment groups had a significant increase in the use of the top at the end of the treatment compared to the control groups (*p* = 0.001 for the 3 mg/L group and *p* < 0.001 for the 5 mg/L group). Furthermore, the 5 mg/L group showed the highest increase in use of the top immediately after treatment compared to both the 3 mg/L (*p* < 0.001) and the control (*p* = 0.004) groups (Figure 4B).

#### Vertical crossings

There was a significant effect of time [χ^2^_(2)_ = 48, *p* < 0.001], in which both treated groups show a significant increase in the number of crossings between the before and after (*p* = 0.05 for the 3 mg/L group and *p* = 0.02 for the 5 mg/L group) times. Additionally, both groups showed a significant increase in crossings between the before and end (*p* = 0.005 for the 3 mg/L group and *p* = 0.002 for the 5 mg/L group) times. Although treatment was not significant, it showed a tendency for significance [χ^2^_(2)_ = 9.07, *p* = 0.08], with buspirone groups showing a tendency for an overall higher number of crossings in comparison to the control fish (Figure 4C).

#### Group cohesion

There were significant effects of treatment [*χ^2^*_(2)_ = 110, *p* < 0.001] and time [*χ^2^*_(2)_ = 23, *p* < 0.001]. Group cohesion generally decreased between the before and after, and the before and the end treatment points for the control (*p* = 0.01 for both), the 3 mg/L (*p* = 0.01 for both) and the 5 mg/L (*p* = 0.01 for both) groups. Furthermore, control fish had a higher degree of cohesion throughout all timepoints compared to the 3 mg/L (*p* < 0.001 for all times) and the 5 mg/L (*p* < 0.001 for all times) groups (Figure 4D).

### Dose response experiment part 2

#### Locomotion

There were significant effects of day [χ^2^_(2)_ = 115, *p* < 0.001], time [χ^2^_(2)_ = 2,018, *p* < 0.001], and the interaction between day and time [χ^2^_(4)_ = 853, *p* < 0.001] on locomotion. There was a significant increase in locomotion between the before and the end times for the day 1 (*p* < 0.001), day 2 (*p* = 0.002) and day 3 (*p* < 0.001) groups. Furthermore, there was also a significant increase between the before and the after time for the day 3 group (*p* < 0.001). Interestingly, fish treated on days 1 and 3 showed higher locomotion levels compared to day 2 at both the after (*p* = 0.004 and *p* = 0.002) and end points (*p* < 0.001 for both). Furthermore, fish treated on day 1 showed the highest locomotion levels at the end point compared to fish treated on day 3 (*p* < 0.001) and day 2 (*p* < 0.001) (Supplementary Figure S1A).

#### Vertical positioning (time spent at the top)

There were significant effects of day [χ^2^_(2)_ = 74, *p* < 0.001], time [χ^2^_(2)_ = 110, *p* < 0.001] and the interaction between day and time [χ^2^_(4)_ = 47, *p* < 0.001]. All groups showed a significant increase in the use of the top of the tank at the end, compared to the before treatment timepoint (*p* < 0.001 for all groups). Notably, only fish treated in day 3 showed a significant increase in the use of the top between the before and the after timepoint (*p* = 0.007). Finally, fish treated on day 1 showed significantly lesser time at the top at the after timepoint, compared to treated fish on days 2 and 3 (*p* < 0.001 for both; Supplementary Figure S1B).

#### Vertical crossings

There was a significant effect of time [χ^2^_(2)_ = 54, *p* < 0.001] and the interaction of time and day [χ^2^_(2)_ = 39, *p* < 0.001] on the number of vertical crossings. However, day [χ^2^_(2)_ = 5, *p* = 0.07] showed no significant effect. Fish showed the most changes in the amount of midline crossings on days 1 (*p* < 0.001) and 3 (*p* = 0.005) between the before and end timepoints. Furthermore, on day 1 there was also a significant difference between the after and end timepoints (*p* = 0.016), but not for day 3 (*p* = 0.59). No significant changes happened on day 2 (Supplementary Figure S1C).

#### Group cohesion

There was a significant effect of day [χ^2^_(2)_ = 10, *p* = 0.008], time [χ^2^_(2)_ = 99, *p* < 0.001], and their interaction [χ^2^_(4)_ = 37, *p* = 0.002] on group cohesion. There was a significant decrease in cohesion between the before and both the after and end times for fish treated on day 1 (*p* < 0.001 for both times) and day 3 (*p* < 0.001 for both times), but not for those treated on day 2 (*p* = 0.87, *p* = 0.15). Cohesion was significantly lowest for fish pre-treatment on day 2, compared to fish on days 1 (*p* = 0.008) and 3 (*p* < 0.001; Supplementary Figure S1D).

### Main buspirone experiment and T0 sampling group

#### Locomotion

There was a significant effect of time [χ^2^_(6)_ = 129, *p* < 0.001] found for locomotion, but treatment [χ^2^_(1)_ = 0.09, *p* = 0.77] had no significant effect. The general trend is that locomotion decreased after bath treatment for all groups (Supplementary Figure S2A).

#### Total acts of aggression

Time [χ^2^_(6)_ = 39, *p* = 0.001] was found to have a significant effect on total instances of aggression, while treatment [χ^2^_(1)_ = 0.79, *p* = 0.38] had no significant effect. Aggression did not vary significantly between treated and control fish, but generally decreased steadily throughout the progression of the experiment for both groups (Supplementary Figure S2B).

#### Undisturbed cohesion

There were no significant effects of time [*χ^2^*_(6)_ = 16, *p* = 0.01] or treatment [χ^2^_(1)_ = 0.04, *p* = 0.85] on cohesion (Supplementary Figure S2C).

#### Cortisol

**Figure 5:**
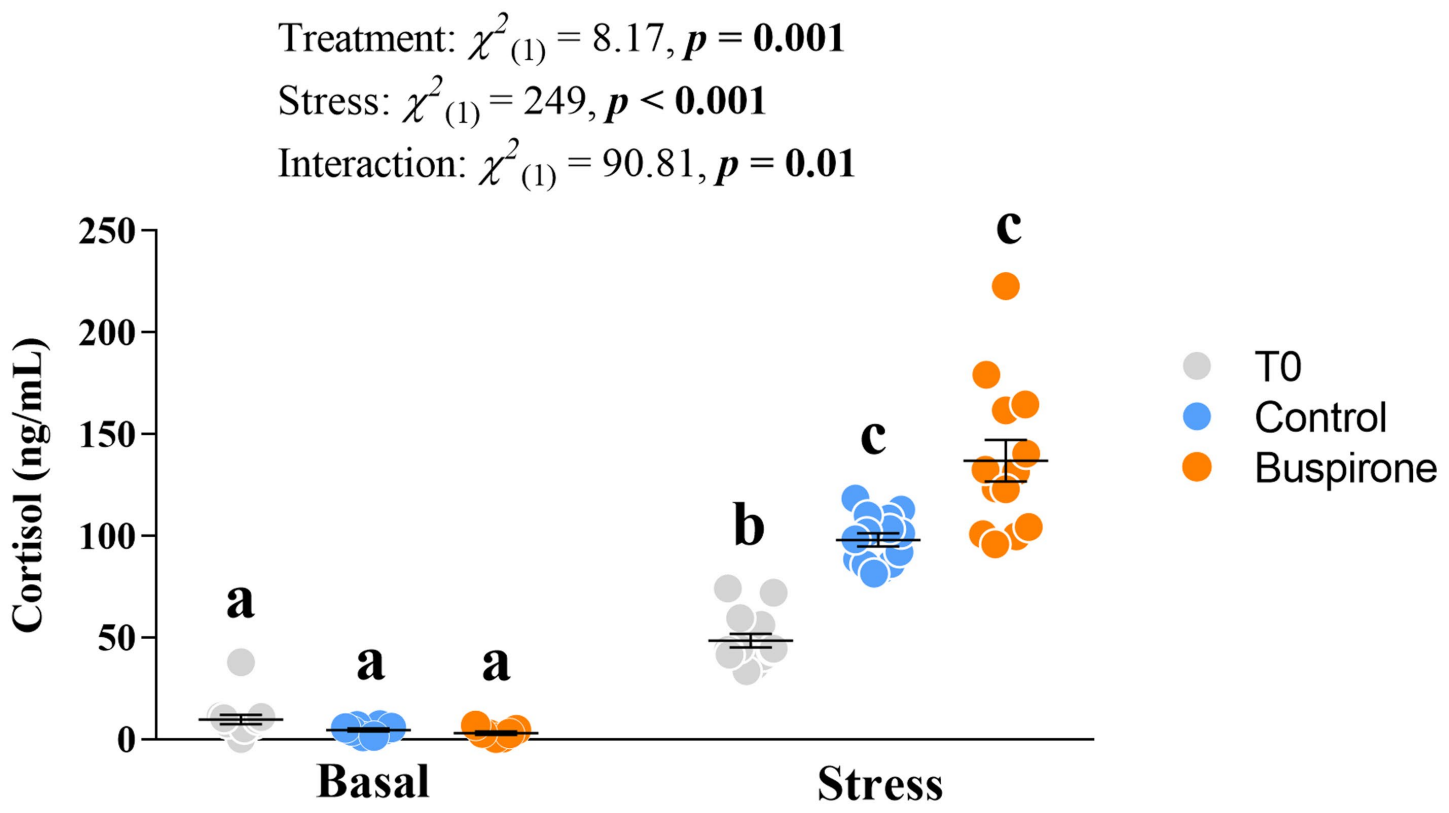
Mean ± SEM plasma cortisol levels of depression-like state (DLS) Atlantic salmon at both basal and post-acute stress conditions for timepoint 0 (not treated in any way), buspirone-treated (i.e., fish were treated twice with 3 mg/L and twice with 5 mg/L buspirone concentrations throughout the course of the experiment) and control (sham-treated) fish. Linear mixed effect model statistics are given within the figure and small letters symbolize Tukey *post-hoc* differences.

There was a significant effect of treatment [*χ*^2^_(1)_ = 8.17, *p =* 0.002], conditions [*χ*^2^_(1)_ = 249, *p* < 0.001] and their interaction [*χ*^2^_(1)_ = 90.8, *p =* 0.01]. Specifically, all groups respond to stress with an increase in cortisol levels (*p* ≤ 0.002 for all), and post-stress levels in the T0 fish were lower than both control (*p* = 0.003) and buspirone (*p* < 0.001) groups (Figure 5).

#### Monoamine neurochemistry

**Figure 6:**
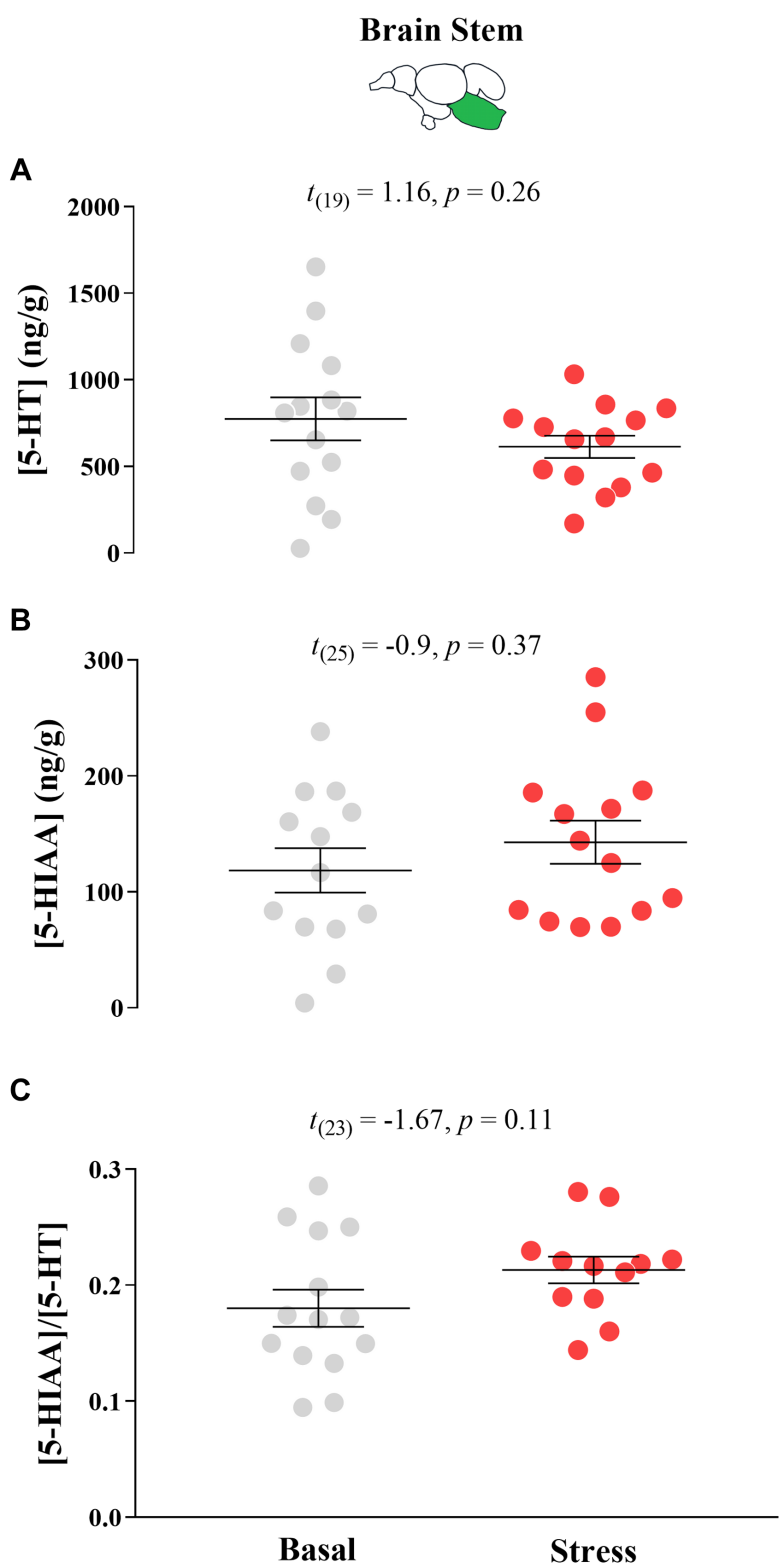
Mean ± SEM serotonin (5-HT) levels **(A)**, its main catabolite 5-hydroxy indole acetic acid (5-HIAA; **(B)** levels, and the 5-HIAA/5-HT ratio **(C)** of growth stunted juvenile Atlantic salmon parr at both basal and post-acute stress conditions at timepoint 0 (i.e., fish were taken from their rearing tank 75 days after sorting). The *t* test statistics are given within each panel.

There were no significant differences in response to acute stress by T0 fish in their 5-HT neurochemistry (Figure 6).

**Figure 7:**
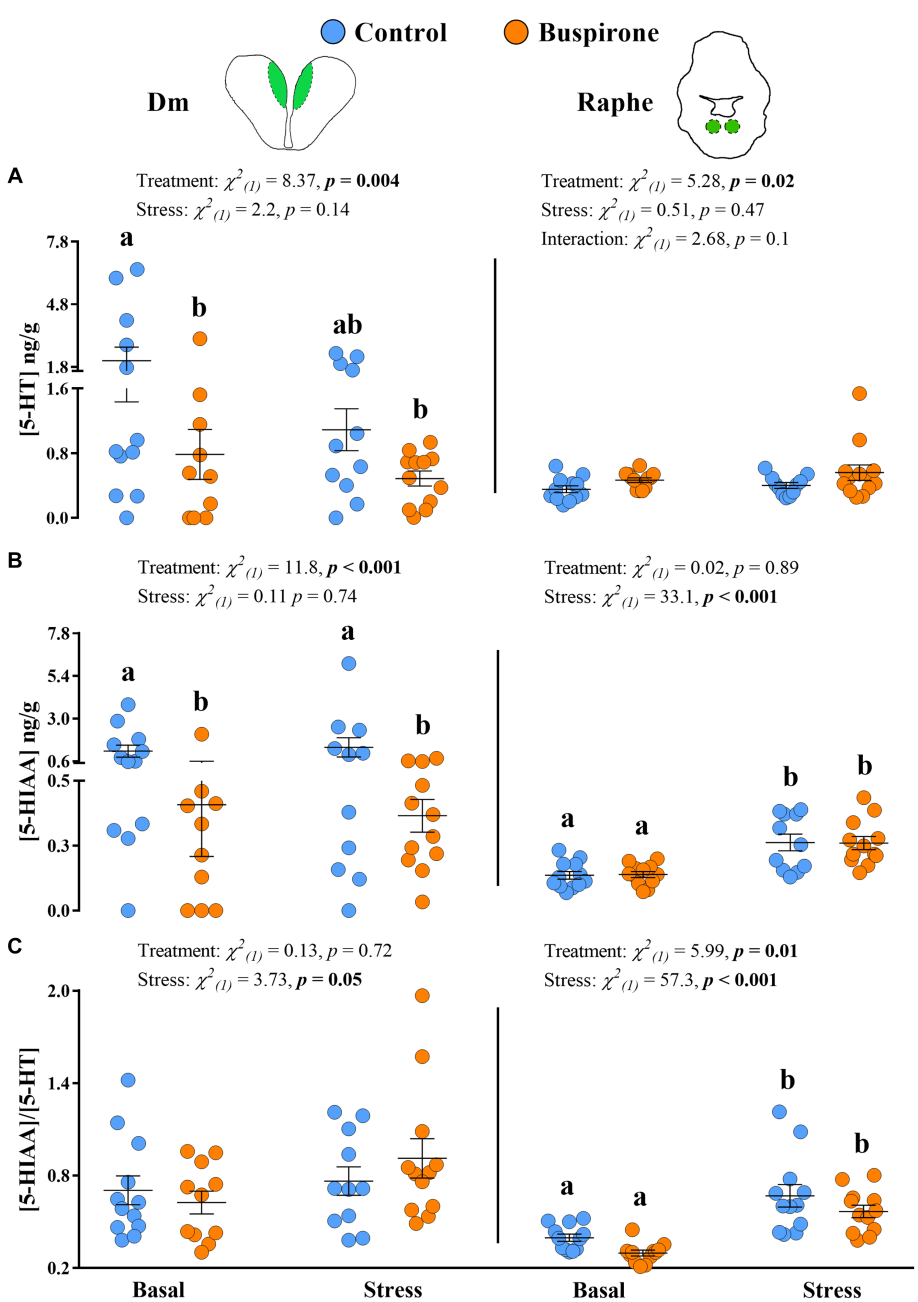
Mean ± SEM serotonin (5-HT) levels **(A)**, its main catabolite 5-hydroxy indole acetic acid (5-HIAA) **(B)** levels, and the 5-HIAA/5-HT ratio **(C)** of depression-like state (DLS) Atlantic salmon at both basal and post-acute stress conditions for buspirone-treated (i.e., fish were treated twice with 3 mg/L and twice with 5 mg/L buspirone concentrations throughout the course of the experiment) and control (sham-treated) fish, in the dorsomedial pallium (Dm; left panel) and the raphe nuclei (RN, right panel). Linear mixed effect model statistics are given within each panel, and lowercase letters symbolize Tukey post-hoc differences.

The 5-HT levels in the Dm showed a significant effect of treatment [*χ*^2^_(1)_ = 7.65, *p* = 0.005], while stress conditions showed no effect [*χ*^2^_(1)_ = 2.02, *p* = 0.15], with the basal control group showing higher levels compared to basal buspirone (*p* = 0.05) and stress buspirone (*p* = 0.02) groups (Figure 7A). Similarly, 5-HIAA levels in the Dm were significantly affected by treatment [*χ*^2^_(1)_ = 10.76, *p* < 0.001] and unaffected by stress conditions [*χ*^2^_(1)_ = 0.07, *p* = 0.78] with buspirone-treated fish showing lower levels compared to controls at both basal and post-stress (*p* = 0.02 for both) levels (Figure 7B). Finally, there were no significant effects of stress [*χ*^2^_(1)_ = 1.27, *p* = 0.25] or treatment [*χ*^2^_(1)_ = 0.30, *p* = 0.57] on the 5-HIAA/5-HT ratio in the Dm (Figure 7C).

In the RN, while treatment [*χ*^2^_(1)_ = 4.56, *p* = 0.03] did show a statistically significant effect on 5-HT levels, stress conditions [*χ*^2^_(1)_ = 0.04, *p* = 0.83] did not, with buspirone groups showing overall higher levels compared to control (Figure 7A). Conversely, treatment [χ^2^_(1)_ < 0.001, *p* = 0.98] did not have a significant effect on 5-HIAA levels in the RN, while stress conditions [*χ*^2^_(1)_ = 35, *p* < 0.001] did. Specifically, there was an increase in 5-HIAA levels in response to stress in both the control (*p* < 0.001) and buspirone (*p* < 0.001) groups (Figure 7B). Similarly, the 5-HIAA/5-HT ratio in the RN was significantly affected by stress conditions [*χ*^2^_(1)_ = 56, *p* < 0.001], showing an increase in response to stress in both the control (*p* = 0.002) and buspirone (*p* < 0.001) groups. Meanwhile, there was a tendency for a treatment effect [*χ*^2^_(1)_ = 3.30, *p* = 0.07], with overall lower activity levels in buspirone groups, compared to controls (Figure 7C).

There were no significant changes in the neurochemistry of the remaining studied areas (Supplementary Table S4).

#### Gene expression

**Figure 8:**
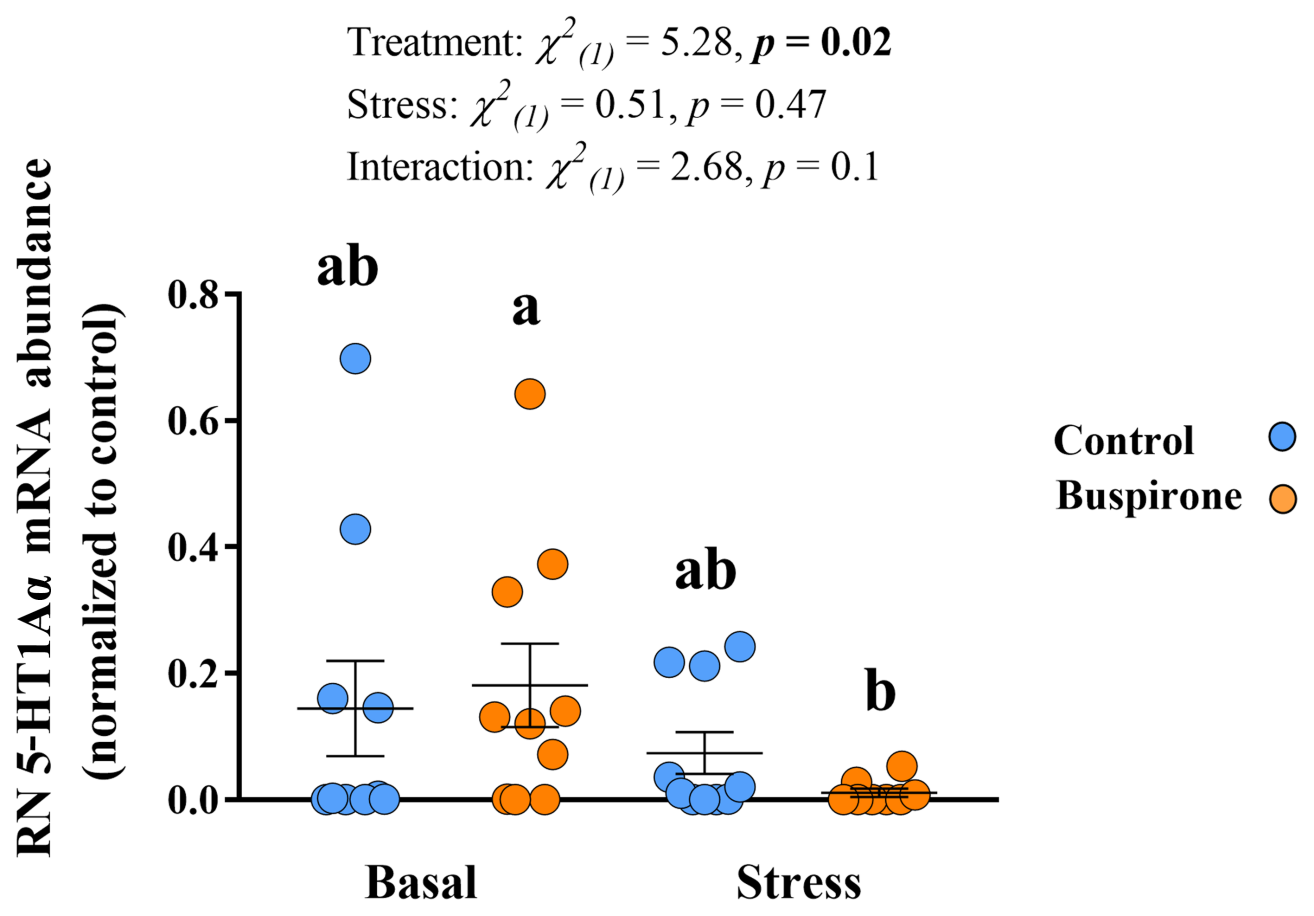
Mean ± SEM relative mRNA abundance (relative to the reference gene, *s20* and normalized to controls) of the serotonin receptor 1Aα (*5-HT1Aα*) in the raphe nuclei (RN) of depression-like state (DLS) Atlantic salmon at both basal and post-acute stress conditions for buspirone-treated (i.e., fish were treated twice with 3 mg/L and twice with 5 mg/L buspirone concentrations throughout the course of the experiment) and control (sham-treated) fish. Linear mixed effect model statistics are given within the figure and lowercase letters symbolize Tukey *post hoc* differences.

In the Dm there were no significant effects in *5-HT_1Aα_* mRNA expression levels for both treatment groups at basal and post-stress conditions (Supplementary Table S4). Meanwhile, there were significant treatment [χ^2^_(1)_ = 7.6, *p =* 0.006], stress [χ^2^_(1)_ = 13.5, *p* < 0.001], and their interaction [χ^2^_(1)_ = 4.87, *p =* 0.03] effects on the RN *5-HT_1Aα_* mRNA abundance levels. Specifically, the buspirone groups responded with a significant decrease in the expression of the *5-HT_1Aα_* compared to basal levels (*p* = 0.01). Furthermore, there was a tendency for buspirone basal to have a higher expression of the *5-HT_1Aα_* gene expression compared to control basal (*p* = 0.08) (Figure 8).

## Discussion

Here, we provide evidence that growth stunted juvenile parr in freshwater show a DLS-like neuroendocrine profile. Furthermore, we show that treating healthy behaviorally inhibited fish with buspirone resulted in less anxiety-like behavior. However, treating DLS-like fish with buspirone resulted in lower basal 5-HT signaling, compared to non-buspirone treated DLS-like fish. While these changes were not accompanied by a measurable change in locomotion, aggression, or cohesion within the experimental timeframe, we believe that the physiological changes are indicative of reversal of the DLS neuroendocrine profile.

The results from our dose response experiment are in accordance with previous results on the effects of buspirone treatment (including a 3 mg/L concentration) in fish (Bencan et al., 2009; Barba-Escobedo and Gould, 2012). In general, we found that fish showing stress-induced behavioral inhibition which were treated with buspirone responded with increased locomotion, higher use of the top part of the tank, and less cohesion. These results are interpreted as individuals showing less anxiety-like behavior (Maximino et al., 2013). Interestingly, we also found that a 5 mg/L buspirone concentration was associated with aberrant behavior (i.e., fish floating with a head up, tail down position and showing muscular spasms) and should not be used on healthy fish.

In our main buspirone experiment on DLS-like fish, we found no quantifiable behavioral effects in response to repeated treatment with either of the 3 or 5 mg/L buspirone concentrations. Buspirone is a prescribed drug with anxiolytic effects, which acts via 5-HT_1A_ receptors. These 5-HT_1A_ receptors are located on dendrites and in the cell bodies of both presynaptic 5-HT neurons, where they mediate the autoregulation of serotonin, and postsynaptic neurons, where they function as heteroreceptors (Albert et al., 2011). Activation of presynaptic 5-HT_1A_ autoreceptors, located on serotonergic neurons in the dorsal and medial RN, results in an inhibition of the firing rate of the serotonergic neurons and thus suppresses 5-HT synthesis (Toth, 2003). On the other hand, released serotonin binds to 5-HT_1A_ heteroreceptors, which exist on dendrites and cell bodies of the postsynaptic neurons. They mediate serotonin-mediated brain-specific responses (Albert and Vahid-Ansari, 2019). Lower serotonin release in the synaptic cleft has an inhibitory effect on postsynaptic 5-HT_1A_ heteroreceptors, which will receive lower doses of 5-HT and further decrease the serotonin release (Toth, 2003). Notably, buspirone has a high affinity for 5-HT_1A_ receptors, where it is a full agonist on presynaptic 5-HT_1A_ receptors and a partial agonist on postsynaptic 5-HT_1A_ receptors (Loane and Politis, 2012). Studies examining the anxiolytic effect of buspirone on rats have determined that buspirone’s efficacy is diminished somewhat at postsynaptic receptors, showing more specificity in blocking presynaptic 5-HT_1A_ autoreceptors (Eison and Temple, 1986; Goa and Ward, 1986). In summary, it has been proposed that buspirone activates autoreceptors to decrease the amount of serotonin in the synaptic cleft, which reduces the serotonergic signaling, resulting in higher exploration and reduced anxiety (Gebauer et al., 2011). However, previous fish research on the effect of buspirone exposure has been conducted only after a single dose (e.g., Maaswinkel et al., 2012; Maximino et al., 2013; Abozaid and Gerlai, 2022) and it is possible that multiple buspirone treatments may affect serotonergic activity in different ways, which may account for the lack of a higher magnitude response in the DLS-like fish. Furthermore, the DLS-like fish, having experienced prolonged chronic stress compared to the short-term stress experienced by healthy fish in the dose response experiment, may have also resulted in a disparity in responses to buspirone treatment. That is, while the dose response experiment fish were experiencing stress as a response to handling, moving, and a novel environment, the DLS-like fish fit a profile of chronic stress that results in traits such as stunted growth, anorexia from lack of feeding, behavioral inhibition, and listlessness (Stien et al., 2013; Vindas et al., 2016, 2019). Fish under continued stress have also been observed to have altered neuroendocrine responses (Barton, 2002; Tort, 2011; Vindas et al., 2019) and suffer from impaired cognitive function and an overall decline in physical health that increases their risk of mortality (Barton et al., 1987; Juell, 1995). This experience of prolonged stress is in fact altering their neurophysiology and they may need either stronger doses or a prolonged treatment time to recover behaviorally from the DLS profile. Similarly, in humans, usage of selective serotonin reuptake inhibitors (SSRIs) as a catch-all treatment for depressive symptoms is not always effective, due to the nature of some depressive states characterized by increased serotonergic activity levels and SSRI’s increasing serotonergic signaling, which effectively leads to a worsening of the depressive symptoms (Andrews et al., 2015).

Here, we categorized the neuroendocrine profile of growth stunted juvenile parr for the first time. We found that when exposed to acute stress, these individuals demonstrate a blunted cortisol response and no 5-HT response. These results are reminiscent of the neuroendocrine profile of DLS fish in seawater (Vindas et al., 2016) and provides evidence of the salmonid DLS phenotype being present early in life during the freshwater stage. For the main buspirone experiment, we aimed at conducting 5-HT neurochemistry analysis in region-specific areas in the brain important for emotional and stress regulation. We found specifically that the dorsomedial pallium (Dm) had lower 5-HT levels at basal conditions for the buspirone group in comparison to the control group. We believe that this is evidence of buspirone working to reduce serotonergic signaling in the Dm region. Importantly, since 5-HT is rapidly replaced intracellularly following its release at the synapse (Shannon et al., 1986) and extracellular 5-HT concentrations reflect both 5-HT release and clearance from the synapse (Andrews et al., 2015), effects of buspirone would then be more evident in both the 5-HIAA levels and the 5-HIAA/5-HT ratio. In agreement with this, we found that 5-HIAA levels in the Dm are also significantly lower in the buspirone group. However, this was not evident in the 5-HIAA/5-HT ratio. Since the formation of 5-HIAA happens after reuptake of 5-HT from the synapse into the presynaptic neuron and both 5-HT and 5-HIAA levels are already lowered by buspirone, a possible effect of buspirone may not be evident in the ratio of these parameters. In fish, the Dm serves as the decision-making and emotional regulation center of the brain, homologous to the amygdala, and contains 5-HT_1A_ heteroreceptors on post-synaptic neurons. The fact that the *5-HT1Aα* relative abundance is increased in the raphe nuclei (RN) of buspirone-treated fish at basal levels, suggests that there is higher activity of this receptor which could result in the Dm decrease of 5-HT synthesis and increased 5-HIAA concentrations. Interestingly, we did not find a significant change in 5-HT or 5-HIAA levels in the RN in response to buspirone treatment. However, there was a tendency for buspirone groups to have lower serotonergic activity at basal levels compared to control fish in this area. Considering that the RN is the main hub for synthesis of 5-HT in the brain (Winberg et al., 1997), lower serotonergic activity in this area would be in accordance with a buspirone effect on autoreceptors and provides further evidence of this treatment effecting 5-HT neurochemistry in treated fish. Altogether, the results on 5-HT neurochemistry and gene expression show that buspirone-treated fish show reduced 5-HT neurochemistry at basal levels, compared to non-buspirone treated DLS-like fish, which is evidence for the reversal of the DLS neuroendocrine profile by buspirone-treatment.

Normally, fish respond to stress with an increase in blood cortisol that naturally decreases over a period of time back to basal levels. However, problems may arise when stress persists over a long period of time, as all of these processes extended over a longer period can result in a reduction of overall fitness and increased susceptibility to illness (Pickering and Pottinger, 1989; Mommsen et al., 1999). The DLS phenotype has been characterized by chronically elevated cortisol at basal levels, which contributes to low weight, behavioral inhibition, and increased mortality rates due to cortisol’s effect on metabolic processes (Sapolsky, 2011). We found that DLS-like fish taken directly from their home tank 75 days after sorting (T0) had a subdued cortisol response to confinement stress. That is, while all treated fish showed a healthy response to stress (increasing 10-fold from basal conditions; e.g., Wendelaar Bonga, 1997), the untreated T0 DLS-like fish showed only a 5-fold increase post-stress, which was significantly reduced compared to both sham-treated control and buspirone-treated fish. This indicates that although these DLS-like fish are able to respond to stress with increased cortisol levels, the magnitude of the response is half that of normal healthy fish. Notably, in the main buspirone experiment all DLS-like fish were essentially granted an environment wherein they had free access to food and no competition or aggression from larger conspecifics (i.e., they were in smaller group of similar-sized fish), both of which are contributing factors to growth disparities and potential emergence of dominance-related disparities between individuals that may lead to DLS profiles (Cubitt et al., 2008; Vindas et al., 2016, 2019). It is therefore possible that we provided an environment in which the DLS-like profile may reverse back to a more normal physiological and behavioral state in both buspirone- and sham-treated fish, which would explain why both these groups showed a healthy 10-fold increase in cortisol to acute stress and similar post-stress 5-HT signaling. This possibility is intriguing and certainly warrants further experiments aiming at changing environmental conditions to elucidate their association with the DLS profile and its reversal.

In conclusion, we found that growth stunted parr, often discarded in commercial aquaculture since they are not big enough to survive vaccination, are characterized by a DLS-like neuroendocrine profile (i.e., blunted cortisol response to acute stress and no response of the 5-HT system). Furthermore, even though buspirone treatment of DLS-like fish resulted in lower basal serotonergic neurochemistry signaling compared to non-buspirone treated fish, no behavioral changes were evident, which suggest that manipulating 5-HT signaling with multiple buspirone treatments may not be an effective way to completely reverse the DLS profile. Instead, a change in environment, effectively removing or diminishing stressors may result in a more dramatic reversal of the DLS profile. This new breadth of information about DLS in salmon can have potential implications for how captive fish are kept in aquaculture and raises questions about the welfare of farmed fish in general. With a deeper understanding of what causes DLS profiles and growth stunting in juvenile fish, steps can be taken in terms of husbandry to prevent repeated stressors and the formation of the DLS profile, potentially reducing losses in aquaculture stock due to chronic stress. Additionally, on a much broader scale, these studies on DLS fish can also inform our understanding of how depressive symptoms are treated in people and the varied results pharmaceutical intervention can have. The outward symptom of depression or a depression-like state is not always tied to the exact same mechanisms and structures in the brain, meaning that drug treatments can result in different outcomes and potentially even worsen symptoms. By studying these fish, conclusions can also be drawn about drug efficacy and repeated treatment effects, as well as the benefits of integrating medical treatment with environmental change which may promote the recovery from DLS in vertebrates.

## Data availability statement

The data sets presented in this study can be found within the Supplementary data files.

## Ethics statement

The animal study was approved by the Norwegian Food Safety Authority. The study was conducted in accordance with the local legislation and institutional requirements.

## Author contributions

SS: Writing – original draft, Writing – review & editing. AS: Writing – original draft. OF: Writing – review & editing. MV: Writing – original draft, Writing – review & editing. TF: Writing – review & editing.

## Footnotes

^1^ http://www.rproject.org
