# Supplementary material for: Behavioral and neurophysiological effects of buspirone in healthy and depression-like state juvenile salmon
Source: Front Behav Neurosci. 2024 Feb 12;18:1285413. doi: 10.3389/fnbeh.2024.1285413 (PMC10894974; doi:10.3389/fnbeh.2024.1285413)
Supplement: Supplementary file 2 [file Data_Sheet_2.docx]

**Behavioral and neurophysiological effects of buspirone in salmon**

Sheyda Shapouri^1^, Aziz Sharifi^2^, Ole Folkedal^3^, Thomas W. K. Fraser^4^, Marco A. Vindas^1^.

^1^ Biochemistry and Physiology Unit, Faculty of Veterinary Medicine, Norwegian University of Life Sciences

^2^ Department of Biosciences, Faculty of Mathematics and Natural Sciences, University of Oslo.

^3^ Animal welfare, Matre Research Station, Institute of Marine Research

^4^ Reproduction and Developmental Biology, Matre Research Station, Institute of Marine Research

**Supplementary material**

**Table 1**: Detailed explanation for images chosen at different times and dates for the cohesion analysis during different daily events (*i.e.* feeding, early morning undisturbed behavior, and bath days).

| **Day** | **Time start** | **Time end** | **Still images** | **Activity** |
| --- | --- | --- | --- | --- |
| Day 9 | 06:19 | 06:29 | 5 (every 2min for 10min) | Undisturbed behavior |
| Day 14 | 06:35 | 06:45 | 5 (every 2min for 10min) | Undisturbed behavior |
| Day 16 | 06:29 | 06:39 | 5 (every 2min for 10min) | Undisturbed behavior |
| Day 17 | 06:38 | 06:48 | 5 (every 2min for 10min) | Undisturbed behavior |
| Day 18 | 06:44 | 06:54 | 5 (every 2min for 10min) | Undisturbed behavior |
| Day 19 | 06:37 | 06:47 | 5 (every 2min for 10min) | Undisturbed behavior |
| Day 20 | 06:40 | 06:50 | 5 (every 2min for 10min) | Undisturbed behavior |

**Table 2.** Nucleotide sequence of primer pairs used for quantitative PCR. Abbreviations: *5-HT_1Aα_: serotonin receptor 1A alpha, 5-HT_1Aβ_: serotonin receptor 1A beta, , s20: ribosomal protein S20, ppia: peptidylprolyl isomerase A, ef-1αa: elongation factor 1αa, hprt1: hypoxanthine phosphoribosyltransferase*, fwd: forward primer, rev: reverse primer

| **Gene** | **Primer Sequence 5’ →3’** | | | **Accession nr.** | **References** |
| --- | --- | --- | --- | --- | --- |
| *5-HT_1Aα_* | Fwd  Rev | ATGCTGGTCCTCTACGGGCG  CGTGGTTCACCGCGCCGTTT | AGKD01067361.1 : 7182-7844* | | Vindas et. al., 2017 |
| *5-HT_1Aβ_* | Fwd  Rev | TTGATCATGCGTTCCCAGCCGA  AAAGGAATGTAGAACGCGCCGA | DY694524.1 | | Vindas et. al., 2017 |
| *s20* | Fwd  Rev | GCAGACCTTATCCGTGGAGCTA  TGGTGATGCGCAGAGTCTTG | NM_001140843.1 | | Vindas et al., 2017 |
| *ppia* | Fwd  Rev | CTATCGGCGATTCCCCCGCC  AACCCCTTGTCCCCGGTGCA | NM_001146606.1 | | Frisk et al., 2020 |
| *ef-1αa* | Fwd  Rev | CCCCTCCAGGACGTTTACAAA  CACACGGCCCACAGGTACA | BT059133.1 | | Vindas et. al., 2017 |
| *hprt1* | Fwd  Rev | CGTGGCTCTCTGCGTGCTCA  TGGAGCGGTCGCTGTTACGG | BT043501.1 | | Vindas et. al., 2017 |

**Table S3.** Removed data outliers from the monoamine neurochemistry and gene expression data of serotonin (5-HT), 5-hydroxy indole acetic acid (5-HIAA) and the 5-HIAA/5-HT ratio in the dorsolateral pallium (Dl), dorsomedial pallium (Dm), the ventral part of the ventral telencephalon (Vv), the preoptic area (POA), and the raphe nuclei (RN). Data outliers were determined by the Rosners test, based on a k = 4

| **Monoamine data** | | |
| --- | --- | --- |
| **Brain regions** | **Monoamines** | **Number of outliers** |
| Dl | 5-HT | 6 |
|  | 5-HIAA | 2 |
|  | 5-HIAA/5-HT | 5 |
| Dm | 5-HT | 4 |
|  | 5-HIAA | 5 |
|  | 5-HIAA/5-HT | 4 |
| Vv | 5-HT | 4 |
|  | 5-HIAA | 8 |
|  | 5-HIAA/5-HT | 2 |
| POA | 5-HT | 9 |
|  | 5-HIAA | 6 |
|  | 5-HIAA/5-HT | 5 |
| RN | 5-HT | 3 |
|  | 5-HIAA | 2 |
|  | 5-HIAA/5-HT | 3 |
| T0 | 5-HT | 0 |
|  | 5-HIAA | 1 |
|  | 5-HIAA/5-HT | 2 |
| **Gene expression data** | | |
| **Brain regions** | **Genes** | **Number of outliers** |
| Dm | *5-HT_1Aα_* | 4 |
| RN | *5-HT_1Aα_* | 13 |

Table 4. Mean (± SEM) concentration of serotonin (5-HT), 5-hydroxyindoleacetic acid (5-HIAA) and their ratio (5-HIAA/5-HT), as well as the relative transcript abundance (to the reference gene *S20)* of the *5-HT receptor 1 A alpha* (*5-HT_1Aα_*) in

buspirone-treated and control (sham-treated) depression-like state (DLS) fish at both basal and acute stress conditions in the dorsolateral (Dl) and dorsomedial (Dm) pallium, the ventral part of the ventral telencephalon (Vv), the preoptic area (POA) and the Raphe nuclei (RN). Linear mixed effect model statistics for treatment (control *vs.* buspirone), conditions (basal *vs.* stress) and their interaction are given for each variable.

| **Area** | **Control** | | | **Buspirone** | | | **LME statistics** | | |
| --- | --- | --- | --- | --- | --- | --- | --- | --- | --- |
|  | **Basal**  **(n=12)** | **Stress**  **(n=13)** | **Basal (n=11)** | | **Stress (n=16)** | **Treatment** | | **Stress** | **Interaction** |
| ***Dl*** |  |  |  | |  |  | |  |  |
| 5-HT | 0.17 ± 0.08 | 0.55 ± 0.25 | 0.59 ± 0.31 | | 0.11 ± 0.04 | *χ^2^_(1)_* = 3.70*, p = 0.54* | | *χ^2^_(1)_* = 4.94, *p = 0.26* | *χ^2^_(1)_* = 8.02, ***p =*** ***0.004*** |
| 5-HIAA | 0.29 ± 0.13 | 0.37 ± 0.14 | 0.33 ± 0.10 | | 0.20 ± 0.07 | *χ^2^_(1)_* = 0.39, *p = 0.53* | | *χ^2^_(1)_* = 0.38*, p = 0.53* | ------------ |
| 5-HAA/5-HT | 0.96 ± 0.27 | 0.85 ± 0.19 | 0.70 ± 0.25 | | 0.74 ± 0.15 | *χ^2^_(1)_* = 1.62, *p = 0.20* | | *χ^2^_(1)_* = 0.09*, p = 0.75* | ------------ |
| ***Dm*** |  |  |  | |  |  | |  |  |
| 5-HT | 2.09 ± 1.13 | 1.09 ± 0.43 | 0.52 ± 0.27 | | 0.49 ± 0.16 | *χ^2^_(1)_* = 7.65*,* ***p = 0.005*** | | *χ^2^_(1)_* = 2.02, *p = 0.15* | ------------ |
| 5-HIAA | 1.18 ± 0.58 | 0.91 ± 0.46 | 0.22 ± 0.10 | | 0.37 ± 0.11 | *χ^2^_(1)_* = 10.76*,* ***p < 0.001*** | | *χ^2^_(1)_* = 0.07*, p = 0.78* | ------------ |
| 5-HAA/5-HT | 0.70 ± 0.16 | 0.76 ± 0.15 | 0.63 ± 0.12 | | 0.74 ± 0.09 | *χ^2^_(1)_* = 0.30*, p = 0.57* | | *χ^2^_(1)_* = 1.27*, p = 0.25* | ------------ |
| *5-HT_1Aα_* | 0.80 ± 0.27 | 1.32 ± 0.62 | 0.62 ± 0.22 | | 1.05 ± 0.48 | *χ^2^_(1)_* = 0.15*, p = 0.69* | | *χ^2^_(1)_* = 0.92*, p = 0.33* | *χ^2^_(1)_* = 0.39, *p = 0.52* |
| ***Vv*** |  |  |  | |  |  | |  |  |
| 5-HT | 0.17 ± 0.08 | 0.10 ± 0.03 | 0.08 ± 0.03 | | 0.16 ± 0.08 | *χ^2^_(1)_* = 0.12*, p = 0.72* | | *χ^2^_(1)_* = 0.01*, p = 0.90* | *χ^2^_(1)_* = 3.72*, p = 0.053* |
| 5-HIAA | 0.03 ± 0.01 | 0.13 ± 0.05 | 0.03 ± 0.01 | | 0.10 ± 0.04 | *χ^2^_(1)_* = 0.541*, p = 0.462* | | *χ^2^_(1)_* = 10.63*,* ***p = 0.001*** | ------------ |
| 5-HAA/5-HT | 0.81 ± 0.26 | 0.82 ± 0.22 | 0.54 ± 0.09 | | 0.74 ± 0.17 | *χ^2^_(1)_* = 2.03*, p = 0.154* | | *χ^2^_(1)_* = 0.63*, p = 0.42* | ------------ |
| ***POA*** |  |  |  | |  |  | |  |  |
| 5-HT | 0.04 ± 0.00 | 0.06 ± 0.02 | 0.04 ± 0.01 | | 0.11 ± 0.04 | *χ^2^_(1)_* *= 2.84,* *p = 0.091* | | *χ^2^_(1)_* = 5.59*, p = 0.018* | ------------ |
| 5-HIAA | 0.02 ± 0.00 | 0.03 ± 0.01 | 0.02 ± 0.01 | | 0.05 ± 0.01 | *χ^2^_(1)_* = 0.861*, p = 0.353* | | *χ^2^_(1)_* = 10.15*,* ***p = 0.001*** | ------------ |
| 5-HAA/5-HT | 0.43 ± 0.07 | 0.56 ± 0.11 | 0.20 ± 0.03 | | 0.69 ± 0.25 | *χ^2^_(1)_* = 0.50*, p = 0.822* | | *χ^2^_(1)_* = 8.81*,* ***p = 0.002*** | *χ^2^_(1)_* = 3.28*, p = 0.069* |
| ***RN*** |  |  |  | |  |  | |  |  |
| 5-HT | 0.35 ± 0.07 | 0.40 ± 0.06 | 0.47 ± 0.05 | | 0.43 ± 0.06 | *χ^2^_(1)_* = 4.56*,* ***p = 0.032*** | | *χ^2^_(1)_* = *0.04, p = 0.83* | ------------ |
| 5-HIAA | 0.14 ± 0.03 | 0.26 ± 0.05 | 0.14 ± 0.02 | | 0.26 ± 0.04 | *χ^2^_(1)_* = *0.0003, p = 0.985* | | *χ^2^_(1)_* = *34.74,* ***p < 0.001*** | ------------ |
| 5-HAA/5-HT | 0.40 ± 0.04 | 0.57 ± 0.06 | 0.30 ± 0.03 | | 0.57 ± 0.07 | *χ^2^_(1)_* = 3.30*, p = 0.069* | | *χ^2^_(1)_* = *56.30,* ***p < 0.001*** | *χ^2^_(1)_* = 2.44*, p = 0.118* |
| *5-HT_1Aα_* | 0.12 ± 0.09 | 0.07 ± 0.04 | 0.34 ± 0.13 | | 0.02 ± 0.02 | *χ^2^_(1)_* = *2.53, p = 0.111* | | *χ^2^_(1)_* = *8.22,* ***p = 0.004*** | *χ^2^_(1)_* = 5.98, ***p = 0.015*** |


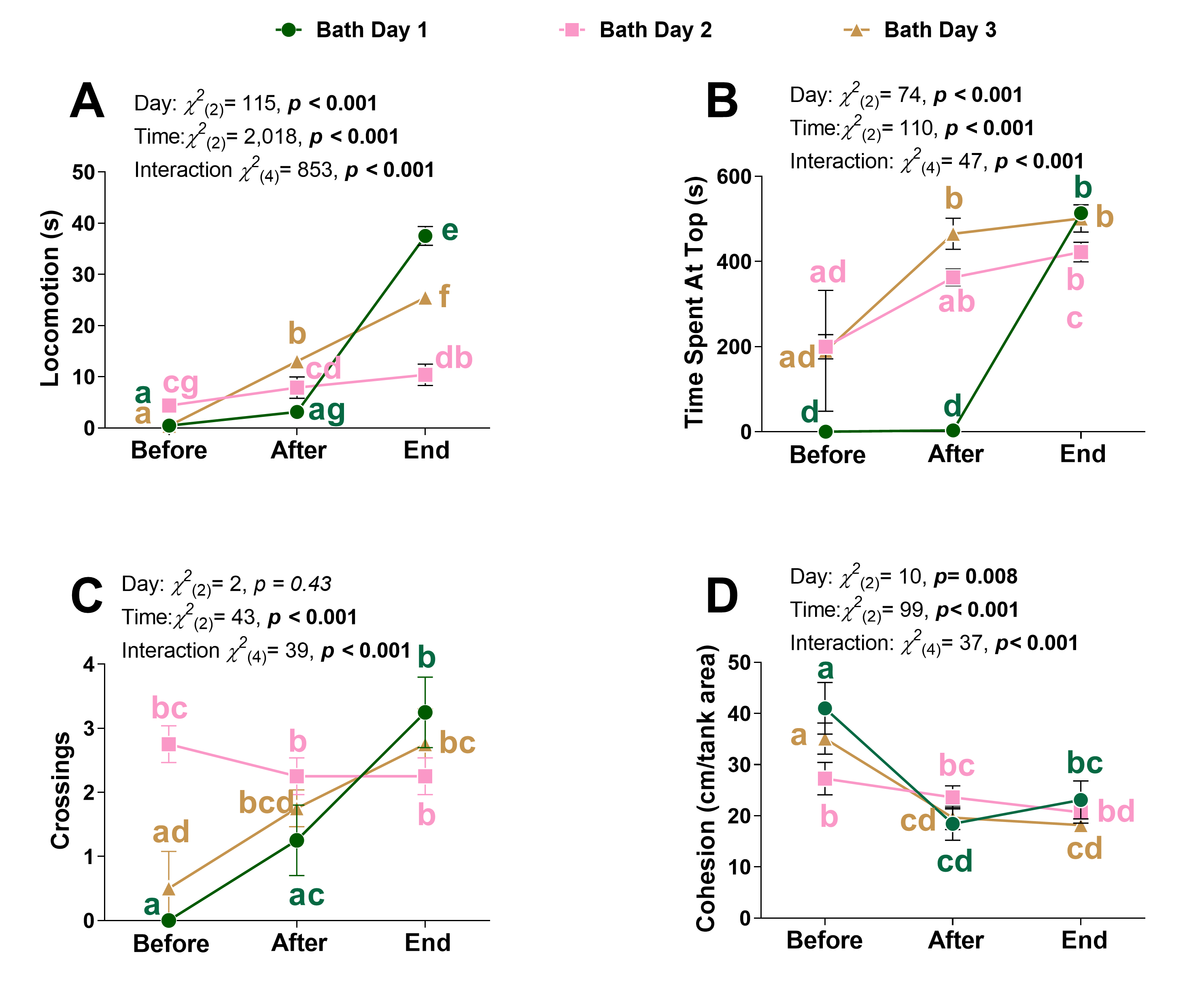


**Figure 1:** Mean ± SEM of locomotion (*i.e.,* time spent moving more than one body length; A), time spent at the top half of the water column (B), the number of times fish crossed between top and bottom halves (C), and overall group cohesion (measured as average distance between all fish within the group; D) of buspirone-treated (3 mg/L) fish. Measurements were taken 10 min before, 10 min after, and at the last 10 min of the buspirone bath which lasted for 1 h. Linear mixed effect model statistics are given within each panel and small letters symbolize Tukey post-hoc differences.


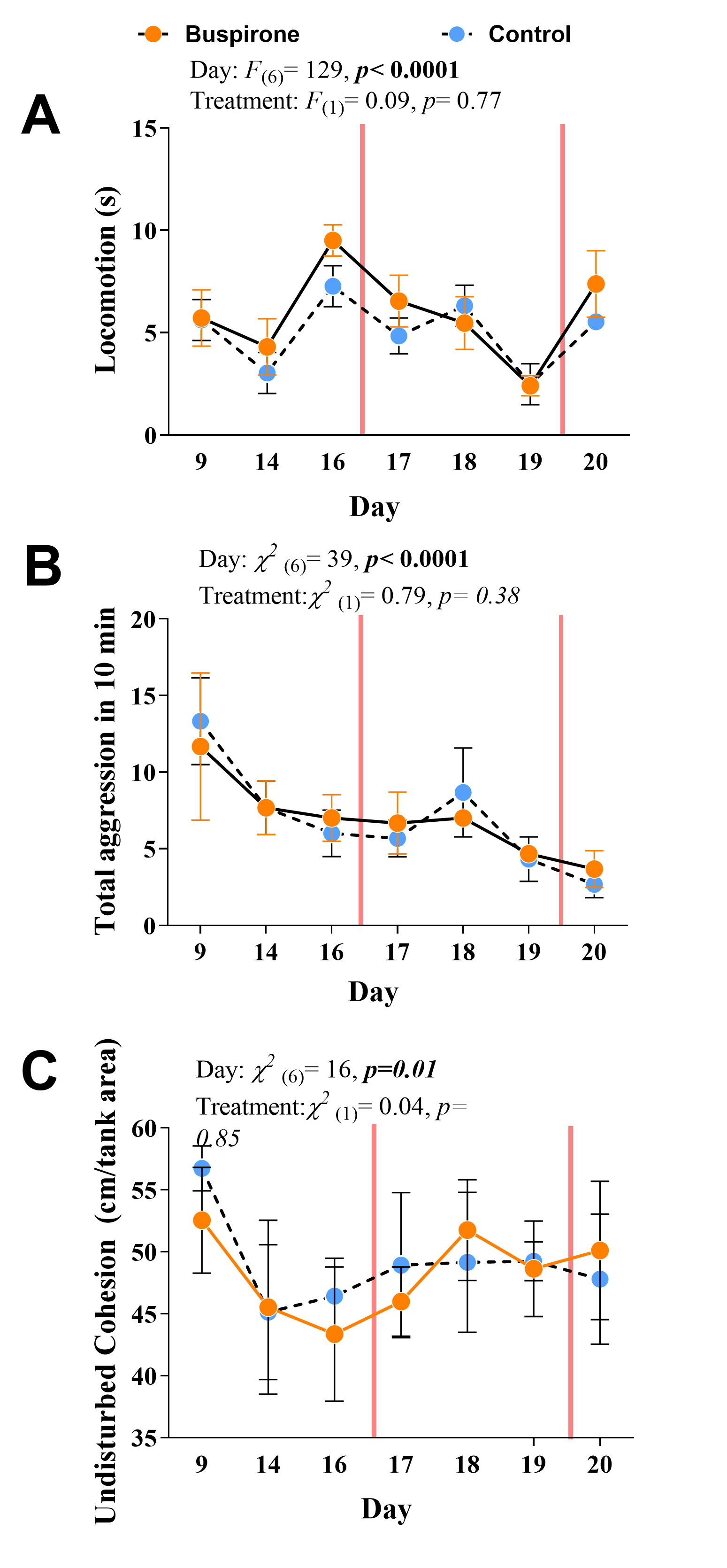


**Figure 2:** Mean ± SEM of anxiety-like parameters measured for buspirone-treated (5mg/L) and control (sham-treated) fish across seven non-consecutive treatment days. The measured parameters were: locomotion (i.e., time spent moving more than one body length; A), total instances of aggression within a 10-minute period (B), and overall group cohesion (measured as average distance between all fish within the group; (C). Linear mixed effect model statistics are given within each panel.
